# Complement activation fragment C5a receptors, CD88 and C5L2, are associated with neurofibrillary pathology

**DOI:** 10.1186/1742-2094-10-25

**Published:** 2013-02-08

**Authors:** Maria I Fonseca, Susan O McGuire, Scott E Counts, Andrea J Tenner

**Affiliations:** 1 Dept of Molecular Biology and Biochemistry, Dept. of Neurobiology and Behavior, Dept. of Pathology and Laboratory Medicine, and Institute for Memory Impairments and Neurological Disorders, University of California, Irvine, 92697, USA; 2 Rehabilitation Research, US Veterans Administration, Edward Hines Jr. VA Hospital, Hines, IL, 60141, USA; 3 Department of Anesthesiology, University of Illinois at Chicago, Chicago, IL, 60612, USA; 4 Department of Neurological Sciences, Rush University Medical Center, Chicago, IL, USA; 5 Department of Molecular Biology and Biochemistry, University of California, 3205 McGaugh, Irvine, CA, 92697-3900, USA

**Keywords:** C5a receptors, CD88, C5L2, Alzheimer’s disease, Complement, Neuropathology, Tangles

## Abstract

**Background:**

Alzheimer’s disease (AD) is a neurodegenerative dementia characterized by the decline of cognition and the presence of neuropathological changes including neuronal loss, neurofibrillary pathology and extracellular senile plaques. A neuroinflammatory process is also triggered and complement activation has been hypothesized to have a relevant role in this local inflammatory response. C5a, a proinflammatory anaphylatoxin generated after complement activation, exerts its chemotactic and inflammatory functions through the CD88 receptor while the more recently discovered C5L2 receptor has been postulated to have an anti-inflammatory role. Previously, we reported that a CD88 specific antagonist (PMX205) decreased the pathology and improved cognition in transgenic models of AD suggesting that C5a/C5aR interaction has an important role in the progression of the disease.

**Methods:**

The present study characterizes the expression of the two receptors for C5a in human brain with confirmed post mortem diagnosis of vascular dementia (VD) or AD as well as age matched controls by immunohistochemistry and Western blot analysis using several antibodies against different epitopes of the human receptors.

**Results:**

The CD88 and C5L2 antibodies revealed increased expression of both receptors in AD samples as compared to age-matched controls or VD brain tissue by Western blot and immunohistochemistry, using multiple antibodies and distinct cohorts of brain tissue. Immunostaining showed that both the C5L2 and CD88 antibodies similarly labeled abundant neurofibrillary tangles, neuropil threads and dystrophic neurites associated with plaques in the hippocampus and frontal cortex of AD cases. In contrast, little or no neuronal staining, tangles or dystrophic neurites associated with plaques were observed in control or VD brains. CD88 and C5L2 receptors are associated with both early (AT8) and mature (PHF1) neurofibrillary tangles and can be found either independently or colocalized with each other.

**Conclusions:**

The observed association of CD88 and C5L2 with neurofibrillary pathology suggests a common altered pathway of degradation.

## Background

Fibrillar amyloid plaques and neurofibrillary tangles are two characteristic lesions observed in Alzheimer’s disease (AD) brains (reviewed in [1]). *In vitro* studies have demonstrated the activation of both classical and alternative complement pathways by fibrillar Aß [2-4]. C5a, a fragment generated by activation of complement, is chemotactic for glia [5]. Therefore, the association of complement components and activation products as well as reactive glia with fibrillar plaques and tangles [6-8] is consistent with the hypothesis that complement activation contributes to and/or exacerbates a local inflammatory reaction around plaques. The detection of C5b-9 associated with dystrophic neurites in plaques and with tangles [9] provides further evidence of complement activation and indicates that C5a anaphylatoxin has been generated as well. Although there are reports of C5a neuroprotective effects [10], increasing evidence suggests that C5a-CD88 interaction has a detrimental effect in neuroinflammatory diseases either directly on neurons [11-14] or indirectly via microglia activation ( [15] and reviewed in [16] and [17]).

The classically described C5a receptor, also identified as CD88, is a G protein-coupled, seven transmembrane-spanning receptor, and its binding to C5a results in intracellular calcium mobilization and activation of several signaling pathways such as MAPK, ERK, DAG and PI3K (for a review see [18,19]). C5L2 is a more recently described C5a binding seven transmembrane receptor that appears to be deficient in G protein coupling. Its function is still not well defined although some evidence indicates that it can be anti-inflammatory (for review see [20]) and/or a modulator of the CD88 mediated signal transduction through the ß arrestin pathway [21]. Expression of CD88 in the periphery has been shown to include not only myeloid but also non myeloid cells such as endothelial and epithelial cells. C5L2 distribution is similarly broad (reviewed in [18]). In the central nervous system (CNS) (human, rat and mouse), CD88 receptor protein and mRNA has been reported to be expressed in astrocytes, microglia, subsets of neurons and neural progenitor cells. While there are more limited studies of C5L2 expression, there is evidence for message in human brain and protein expression in rat astrocytes and subsets of neurons (reviewed in [22]). However, the role of either receptor in neurons is not well established and likely to be complex depending on the environmental signals as reviewed by Woodruff and colleagues [22].

Studies with mouse models of AD also show the association of complement factors and receptors with amyloid plaques [23,24] demonstrating complement activation in those models. More recently, our observation of a neuroprotective effect of a CD88 antagonist [25] in two mouse models of AD added support to the hypothesis that the consequences of C5a-CD88 interactions are detrimental in the aging brain. The effect of specific C5aR antagonists in decreasing the pathology and improving clinical symptoms in animal models of other neurodegenerative diseases [26,27] suggests that C5a-CD88 interaction accelerates disease and can be a therapeutic target. In contrast, there is evidence suggesting that C5L2 receptors have an anti-inflammatory function [28-30].

Reports of the relative expression of CD88 in neurons in AD are conflicting. O’Barr *et al*. observed comparable expression in neuron populations of AD and normal control brains [12], while Farkas and colleagues reported that the neuronal expression of CD88 is decreased in AD, although it was seen to be present in dystrophic neurites associated with plaques [31]. At present, there are no reports of the C5L2 distribution in the AD brain. Here we report the expression of both C5a receptors in cases of vascular dementia (VD) and AD as compared to age-matched controls using several antibodies against different epitopes of the receptors. These data show that CD88 and C5L2 protein levels are increased in AD and that these receptors are associated with AD neurofibrillary pathology (neurofibrillary tangles, dystrophic neurites associated with plaques and neuropil threads).

## Material and methods

### Subjects

Human brain tissue, including 5 VD, 11 AD and 9 age-matched controls, was obtained from the Tissue Repository of the Institute for Memory Impairments and Neurological Disorders at the University of California, Irvine (UCI). Cases studied are shown in Table 1. Tissue was collected within 1 to 13 hours of death and was fixed in 4% paraformaldehyde or 10% formalin (used for immunohistochemistry (IHC)) or frozen (used for Western blots). This study also included 24 cases from the Rush Religious Order Study [32] with *ante mortem* clinical diagnoses of either no cognitive impairment (NCI) (n = 12, Mini-Mental State Examination (MMSE) Score = 27.6 +/− 1.4 (mean +/− SD)) or mild/moderate AD (n = 12, MMSE = 19.9 +/− 6.4). The Braak tangle stage was: NCI:3.0 +/− 1.35, AD:3.75 +/−1.14. The cases were matched for age (total = 85.6 +/− 5.1 years) and post mortem interval (total = 5.1 +/− 2.5 hours) [32]. Hippocampal samples from each case were dissected and snap-frozen at −80°C until used for Western blot. All the tissue obtained from Alzheimer Disease Centers at UCI or Rush was acquired under approved Institutional Review Board protocols.

**Table 1:** List and information of cases studied

**Case year**	**Case num**	**Age**	**Sex**	**MMSE**	**PMI**	**APOE**	**NPDx1**	**NPDx2**	**Braak tangle stage**	**Braak plaque stage**
2003	9	81	M	27	6.4	2/3	N/MBC		II	0
2006	34	79	M	N/A	3.52	2/4	N/MBC	Other	V	B
2005	32	89	F	24	3.58	3/3	N/MBC		III	B
2004	30	90	F	30	3.8	3/3	N/MBC	Other	IV	B
2005	12	78	M	12	2.8	3/4	N/MBC	DLBD	III	B
2005	13	94	F	N/A	5.92		N/MBC		I	A
1997	27	66	M	N/A	13.37	N/A	N/MBC		N/A	N/A
1997	32	68	M	N/A	2.43	N/A	N/MBC		N/A	N/A
1999	10	70	M	N/A	5.2	N/A	N/MBC		N/A	N/A
2004	8	75	F	N/A	10.5	3/3	N/MBC	VD	II	0
2003	4	83	M	28	12.4	4/4	N/MBC	VD	III	0
2002	17	74	M	14	2.6	3/4	N/MBC	VD	II	A
2001	20	85	M	15	3.2	2/4	N/MBC	VD	III	0
2001	14	73	M	N/A	4.3	3/3	N/MBC	VD	0	0
2006	1	63	M	10	5.8	3/4	AD		VI	C
2006	21	82	M	16	4.7	3/4	AD		VI	C
2004	10	71	M	3	6.4	3/3	AD		VI	C
2004	44	96	F	N/A	4.3	3/4	AD		VI	C
2004	46	94	F	N/A	26.92	2/3	AD		VI	C
2005	31	79	M	6	6	3/4	AD		VI	C
2007	15	75	M	18	5.08	3/4	AD		VI	C
2008	3	72	M	0	8.25	4/4	AD		VI	C
2008	4	68	M	3	4.83	3/4	AD		VI	C
2001	12	61	F	N/A	2.8	3/4	AD		VI	C
2001	18	74	F	N/A	4.5	3/4	AD		VI	C

AD, Alzheimer’s disease; MMSE, score for the Mini Mental Status Exam; N/A, not available; N/MBC, normal mild Braak changes; NPDx1, neuropathological diagnosis; NPDx2, secondary neuropathological diagnosis; PMI, post mortem interval in hours; VD, vascular dementia.

### Immunohistochemistry

Tissue sections (50 um) (three to five per case from hippocampus or cortex) were immunostained, as described previously [33]. Briefly, sections were pretreated with 3% H_2_O_2_/10% MeOH/Tris buffered saline (TBS), pH 7.4 to block endoperoxidase. For staining with antibodies that needed antigen retrieval pretreatment, antigen unmasking solution was used following the manufacturer’s instructions (Vector, Burlingame, CA, USA). After blocking with 2% BSA/10% normal goat serum/0.3%Triton/TBS, sections were incubated with the corresponding antibodies (Table 2) in blocking solution, overnight at 4°C. For C5L2 staining, the following antibodies were used: rabbit anti C5L2 N–terminal peptide 1–23 (4 ug/ml, Hycult Biotechnology, Uden, The Netherlands), anti C5L2 N–terminal peptide 1–50 (5 ug/ml, Abcam, Cambridge, MA, USA), anti C5L2, aa248-311 (Abcam), and anti C5L2 C-terminal peptide (aa 275–325) (5 ug/ml, Novus Biologicals, Littleton, CO, USA), and mouse anti C5L2 clone 4C8 (5 ug/ml, gift of Dr. T. Woodruff, raised against human C5L2 transfected murine pre B L1.2 cells (Bamberg *et al*., 2010)) and anti C5L2 clone1D9M12 (Biolegend, San Diego, CA, USA). For CD88, rabbit anti CD88 C-terminal peptide 300–350 (5 ug/ml, Novus), anti C-terminal peptide around phosphorylation site (ser338) and anti CD88 N-terminal peptide (both 5 ug/ml, Abcam), as well as the monoclonal rabbit CD88 clone C85-2506 (BD Biosciences, San Jose, CA, USA) and mouse anti CD88 N term1-31,clone S5/1 (Serotec, Raleigh, NC, USA) were used. After incubation with primary antibody overnight, sections were incubated with biotinylated anti rabbit or anti mouse antibodies (Vector, Burlingame, CA, USA) (1 hour at room temperature) followed by Vectastain ABC (Vector) (1 hour at room temperature) and developed with DAB (3-3’diaminobenzidine) substrate kit (Vector). Tissue was dehydrated and mounted with DePeX (BHD Laboratory Supplies, Poole England).

**Table 2:** Antibodies tested in the study

**Antibodies**	**Type and source**	**IHC**	**WB**
C5L2 N 1-23	Rabbit polyclonal (Hycult)	+	+
C5L2 N 1-50	Rabbit polyclonal (Abcam)	+	+
C5L2 C-term peptide	Rabbit Polyclonal (Novus)	+	–
C5L2 248-311	Rabbit Polyclonal (Abcam)	–	+
C5L2 clone 1D9M12	Mouse monoclonal (Biolegend)	–	+
C5L2 clone 4C8	Mouse Monoclonal (T.Woodruff)	+	ND
CD88 C 300-350	Rabbit polyclonal (Novus)	+	–
CD88 N term peptide	Rabbit polyclonal (Abcam)	+	ND
CD88 C term peptide	Rabbit polyclonal (Abcam)	+	+
CD88 clone C85-2506	Rabbit monoclonal (BD Pharmingen)	–	+
CD88 clone S5/1 N1-31	Mouse monoclonal (Serotec)	–	ND

IHC, immunohistochemistry; ND: not done; WB, Western blot.

For immunofluorescent colocalization of the CD88 or C5L2 receptors with markers of hyperphosphorylated tau (AT8, PHF-1), sections were incubated first with the anti CD88 or C5L2 antibody (overnight at 4°C), followed by the corresponding Alexa 555 labeled anti rabbit secondary antibody (Invitrogen, Molecular Probes, Eugene, OR, USA). After washes and a blocking step the corresponding second primary antibody (AT8 1:50, Pierce, Rockford, IL, USA or PHF-1 1:1000, a gift from Dr. P. Davies, Albert Einstein College of Medicine, Bronx, NY) was added (overnight at 4°C) and developed with Alexa 488 labeled anti mouse secondary antibody (Invitrogen, Carlsbad, CA, USA). Tissue was mounted with Vectashield (Vector).

Colocalization of CD88 and C5L2 was done by sequential staining using first N1-50 C5L2 antibodies (overnight incubation) followed by Alexa555 anti rabbit secondary antibody (1 hour, 4°C). After washing and blocking with normal rabbit serum for 30 minutes (to saturate possible free sites of the secondary antibody), anti CD88 antibody (Novus C300-350), fluorescently labeled with fluorescein isothiocyanate (FITC) using the Lightning link antibody labeling kit (Novus Biologicals) following the manufacturer’s instructions, was added and incubated overnight at 4°C.

Specificity of staining was demonstrated by a lack of reactivity when using rabbit immunoglobulin G (IgG; at the same concentrations as the primary antibodies) or no antibody instead of primary antibody plus the secondary antibodies as controls. In addition, anti C5L2 (N1-23) (5 ug/ml) was preabsorbed with 100 ug/ml C5L2 (N1-23) (United Peptide, Bethesda, MD, USA) and then used to validate the specificity of staining in IHC. Pictures were taken under bright field or fluorescence optics with Zeiss Axiovert 200 (Carl Zeiss, Thornwood, NY, USA). Images were captured with Zeiss Axiovision 4.6 software. Confocal images were obtained with Zeiss LSM 510 confocal microscope. To evaluate the extent of colocalization between pairs of images of C5L2 (red channel) and CD88 (green channel), the Mander’s coefficient was calculated using ImageJ software with JaCoP plugin [34]. The values of Mander’s coefficients range from 0 to 1, with 1 indicating 100% colocalization and 0 indicating no-colocalization.

### Western Blot

Hippocampi were homogenized (10 to 12 strokes with a glass homogenizer) in 2% SDS TBS (50 mM Tris–HCl buffer, 150 mM NaCl, pH 7.4) (0.150 g tissue/ml), containing a cocktail of protease inhibitors (complete Mini) (Roche Diagnostics, Indianapolis, IN, USA). Homogenates were centrifuged at 4°C at 15,000 g for 30 minutes. Protein concentration in the supernatants was determined with the BCA protein assay (Pierce, Rockford, IL, USA). Samples (40 ug protein per lane) were run on 10% SDS polyacrylamide gel under reducing conditions. Proteins were transferred to polyvinylidene difluoride (PVDF, Millipore Corporation, Bedford, MA, USA) membranes (300 mA for 2.5 hours). Membranes were blocked with 3% dry milk in 0.1% Tween/TBS for 1 hour and then incubated for 2 hours at room temperature with primary anti C5L2 or CD88 antibodies (Table 2). In some cases, C5L2 N1-23 antibody (2 ug/ml) with or without a preincubation with the blocking peptide N1-23 peptide was used as the probe. After washing, blots were incubated with horseradish peroxidase (HRP)-labeled anti rabbit or anti mouse secondary antibodies (1:7500) for 1 hour. Labeling was detected using the ECL system (GE Healthcare, Pittsburg, PA, USA). Blots were treated with stripping buffer (100 mM 2-mercaptoethanol, 2% SDS, 62.5 mMTris-HCl, pH 6.7) following the manufacturer’s instructions (Amersham) (and verified to lack any residual reactivity) and subsequently labeled with anti CD88 antibodies (Table 2) (C terminal Abcam or C terminal Novus, 2 ug/ml, or clone C85-2506, BD 1.5 ug/ml), followed by anti rabbit HRP-labeled secondary antibodies (1:7500 dilution, Jackson Immunoresearch,West Grove, PA, USA) and detected with ECL. Finally, blots were stripped and probed with anti ß actin antibody (1:10,000 dilution, Sigma, St. Louis, MO, USA) followed by anti mouse HRP-labeled secondary antibody (1:10,000 dilution, Jackson) and detected with ECL. Quantification of the bands was done using Image J (National Institutes of Health) software. Each sample was tested in two Western blots in independent experiments. Data were analyzed using Student *t*-test statistical analysis.

Alternatively, samples from the Rush Religious Orders Study were prepared as previously reported [32]. Briefly, frozen hippocampi were homogenized with protease inhibitors by sonication for 10 seconds and centrifuged at 100 g for 10 minutes at 4°C. Supernatant proteins (25 ug/sample) were separated by SDS-PAGE, transferred to Immobilon P membranes (Millipore), blocked in TBS (pH.7.4)/0.1% Tween-20/5% milk and incubated overnight at 4°C with rabbit polyclonal C5L2 antiserum (aa248-311) (5 ug/ml Abcam #96808). Blots were incubated for 1 hour with HRP-conjugated goat anti-rabbit IgG. The blots were then probed overnight with a mouse monoclonal beta-actin antibody (1:20,000; Millipore, Billerica, MA, USA). Reactivity was quantified using Kodak one-dimensional image analysis software (Perkin-Elmer, Waltham, MA, USA). C5L2 protein levels were normalized to β-actin levels in each sample. Each sample was analyzed on three different Western blots in independent experiments. Data were analyzed using Student *t*-test. Correlation analysis between C5L2/ßactin ratio and Braak tangle stage for UCI and Rush samples was done using the Spearman rank test.

## Results

### C5L2 distribution in control, vascular dementia and Alzheimer’s disease brain

**Figure 1:**
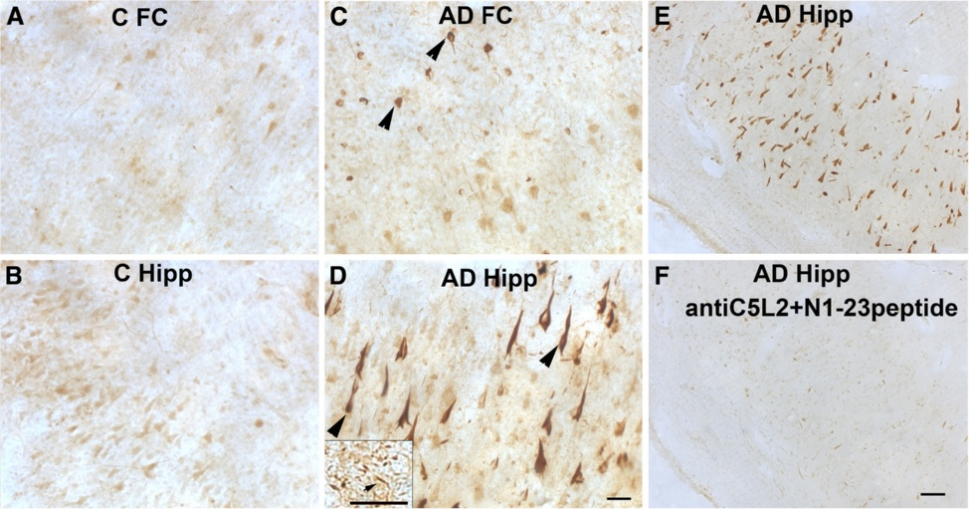
**C5L2 is present in neurons of control brain and neurofibrillary tangles of AD brain.** Immunostaining with anti C5L2 antibody N1-23 shows labeling of a subset of neurons in frontal cortex (FC) (**A**) and hippocampus (**B**) of control cases and strong staining of tangles (arrowheads) in frontal cortex (**C**) and hippocampus (**D**) of AD cases. Scale bar: 50 um. Arrow in insert (**D**) shows neuropil threads. C5L2 N1-23 antibody immunostaining in hippocampus of an AD case (**E**) disappears after antibody preabsorption with the peptide (**F**). Scale bars: A,B,C,D (and insert): 50 um; E,F: 100 um. AD, Alzheimer’s disease.

**Figure 2:**
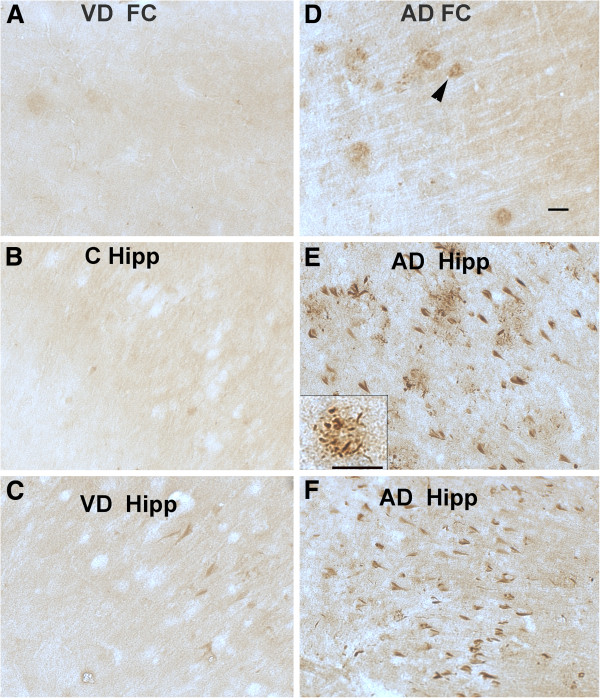
**C5L2 antibodies label dystrophic neurites associated with plaques as well as neurofibrillary tangles.** Representative pictures of C5L2 staining using antibody N1-50 in FC of VD (**A**) and hippocampus of control (**B**) and VD (**C**) brain, and FC (**D**) or hippocampus of two AD cases (**E, F**). Arrowhead in D shows dystrophic neurites associated with plaques in cortex (DNP). Insert in E shows DNP in hippocampus. Scale bars: 50 um. AD, Alzheimer’s disease; FC, frontal cortex; VD, vascular dementia.

The distribution of C5L2 receptor in the brains of control (n = 9), AD (n = 11) and VD (n = 5) cases was assessed by IHC using three polyclonal antibodies, two against the extracellular N terminus of the receptor and one against the intracellular C terminus domain. N1-23 antibody showed a light reactivity with what appeared to be healthy neurons (mainly pyramidal) in frontal cortex and hippocampus of controls (Figure 1A,B) and VD cases (data not shown). In hippocampus of some of these cases a few neurofibrillary tangles (NFT) were stained (data not shown). However, in AD brain, the antibodies strongly labeled a subset of mainly intraneuronal NFTs in frontal cortex (Figure 1C) in 9 of 11 cases, and more abundantly in the CA area (mainly in the CA1 and CA2 subfields) of hippocampus (Figure 1D and Additional file 1: Figure S1). Some neuropil threads (NT) were also labeled (Figure 1D, insert). C5L2 antibody N1-23 immunoreactivity in frontal cortex or hippocampus was blocked by preabsorption of the antibody with the N1-23 peptide (Figure 1E and F) showing specificity of the anti-peptide antibody. The antibodies against the N1-50 region of C5L2 showed immunoreactivity in scarce dystrophic neurites associated with plaques (DNP) in frontal cortex of some controls and VD cases (Figure 2A and data not shown). In hippocampus, these cases were either negative (Figure 2B) or showed very few DNP or tangles labeled (Figure 2C). In contrast, in the AD brains, similar to the N1-23 antibody, NFT and NT were strongly immunoreactive with the N1-50 antibody (Figure 2E, F) in hippocampus, although not in frontal cortex. However, N1-50 abundantly stained DNP in frontal cortex (Figure 2D) and in hippocampus (Figure 2E, and insert).

Another antibody against the carboxy terminal region of C5L2 (Novus), labeled DNP that were very abundant in the AD brain compared to the control and VD cases (data not shown), similar to the pattern of the N1-50 antibody. In addition, this antibody showed some light neuronal staining and reacted also with endothelial cells of blood vessels in both controls and AD (data not shown).

Other anti C5L2 antibodies were also tested. A monoclonal antibody clone 4C8 [21]) showed light neuronal staining that was similar in control and AD cases (data not shown). No immunoreactivity was detected using polyclonal antibody against 248–311 or clone 1D9M12 monoclonal anti C5L2 (data not shown). These data indicate that the epitopes recognized by these antibodies are not accessible in these fixed tissue samples.

**Figure 3:**
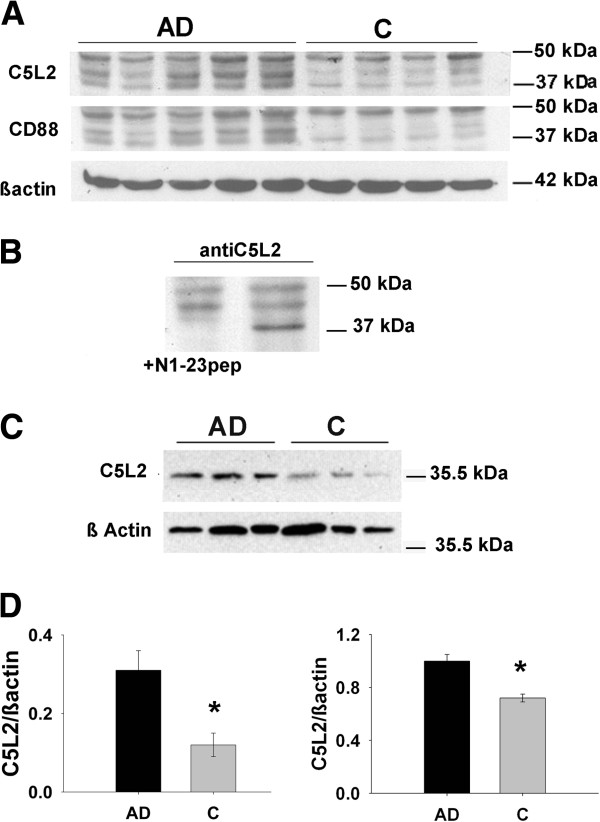
**C5L2 and CD88 expression is upregulated in AD cases. A**. Control and AD extracts from hippocampus subjected to SDS polyacrylamide gel and transferred to PVDF membranes were probed sequentially with anti human C5L2 antibody (N1-23 epitope) upper lane, anti human CD88 (Abcam, C terminal epitope) (middle lane) and anti ß actin (lower lane). **B**. Western blot of hippocampus lysates of AD case probed with C5L2 N1-23 antibody with (left lane) and without (right lane) preadsorption of the antibody with the N1-23 peptide. The 37 k Mr band is eliminated by preadsorption. **C**. Western blot of hippocampus homogenates of control and AD cases (from the Rush Religious Order Study) using Abcam polyclonal antibody against aa248-311of C5L2 and ß actin antibody for further densitometric quantification. **D**. Densitometric quantification of C5L2 immunoblots. Bars show average of ratio of C5L2/ßActin +/− SE from Figure 3A which are AD (n = 5) 0.31 +/−0.05 and C (n = 4) 0.12+/− 0.03, *P* <0.01 (left) or from Figure 3C and data not shown AD (n = 12) 1.05+/−0.05 and C (n = 12) 0.72+/− 0.03, *P* <0.01 (right). AD, Alzheimer’s disease; C, control; n, number; PVDF, polyvinylidene difluoride.

The expression of C5L2 was assessed in SDS lysates from hippocampus of the same control and AD cases used for IHC by Western blot analysis. Both N1-23 and N1-50 antibodies showed immunoreactivity with three bands at 50 k, 45 k and 37 k Mr (Figure 3A and data not shown). No other bands were observed in the blot. The bands at 45 k Mr (expected for the native glycosylated form of the C5L2 receptor) and 37 k Mr (expected size of the receptor based on protein sequencing) were stronger in all the AD cases tested than in control cases where they were almost undetectable relative to comparable protein load as validated with actin labeling (Figure 3A). Densitometric quantification of the 37 k Mr band normalized to actin showed a statistically significant 158% (control, n = 4; AD, n = 5; *P* <0.01) increase in the AD samples (Figure 3D). The 50 k Mr band was variable among the control and AD cases with no clear differences between the groups. Probing with irrelevant rabbit IgG gave no such bands. Preabsorption of the C5L2 antibody with the peptide N1-23 (100 ug/ml) eliminated completely the 37 k Mr band (Figure 3A, 3D left panel) validating the specificity of the antibody for the peptide in solution. The 45 k Mr band was not included in the Western blot quantification since its reactivity was not reduced by preabsorption with the peptide. Although the 45 k Mr band could be a glycosylated state of the receptor, we could not confirm the specificity of that band.

These Western blot results were corroborated using control (n = 12) and mild AD (n = 12) hippocampal homogenates from a different source and probing with a different anti human C5L2 antibody (anti C5L2 aa248-311, Abcam, Table 2). Densitometric quantification of the Western blot membranes of those samples similarly showed a significant increase (46%, *P* <0.01) in the 37 k Mr band of the AD relative to the control samples (Figure 3C, 3D right panel). To corroborate these results further, lysates from the UCI cases were tested with the Abcam antibody used in the Rush cases. Similar to the results shown in Figure 3C, only one band at 37 k Mr was detected, further demonstrating that the 37 k Mr band is C5L2 (Data not shown).

A Spearman correlation analysis between the C5L2/ß actin ratio and Braak tangle stage indicated a positive correlation for both UCI (r = 0.8, *P* <0.01) and Rush cases (r = 0.4, *P* <0.06). In the UCI cohort the positive correlation was strong and reached statistical significance since all the AD cases were late stage in contrast with the Rush cohort that included mostly mild to moderate AD cases.

In summary, C5L2 receptors are mainly detected associated with neurofibrillary pathology and are more abundantly expressed or accumulated in AD brain correlating with Braak tangle stage.

### CD88 distribution in control, vascular dementia and Alzheimer’s disease brain

**Figure 4:**
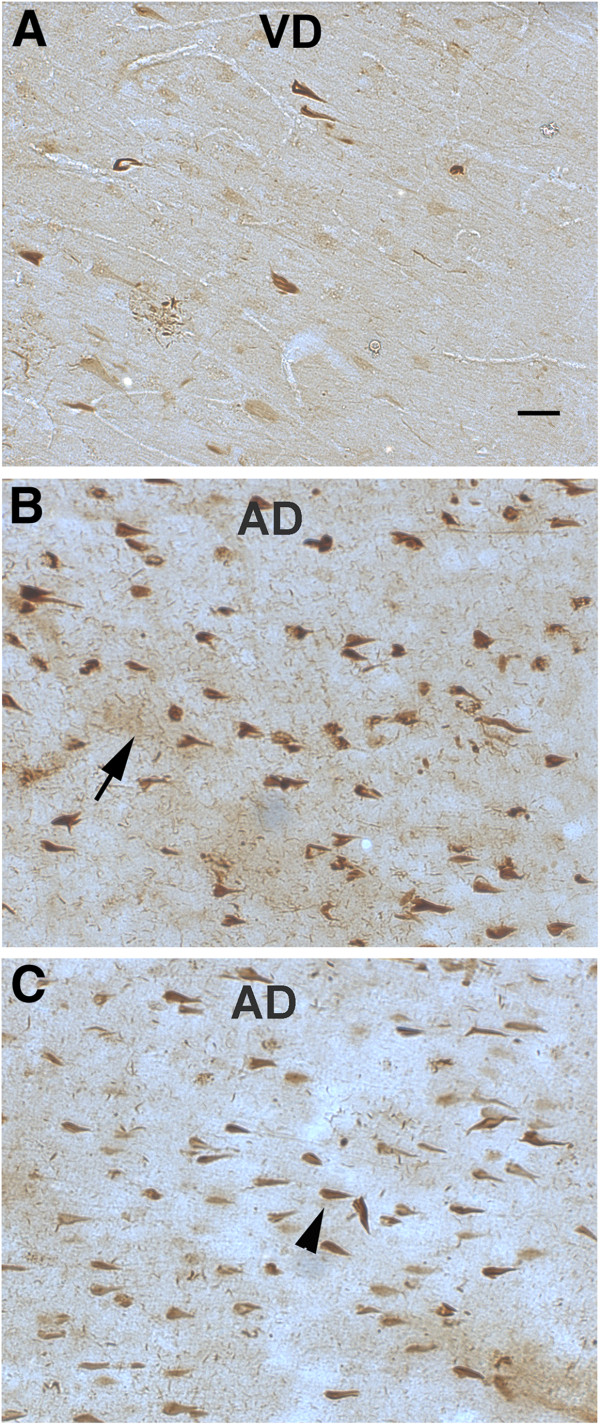
**CD88 immunoreactivity is also abundantly present in neurofibrillary tangles of AD cases.** Immunostaining using two antibodies against different CD88 epitopes: Novus carboxy terminal (**A**, **B**) and Abcam amino terminal (**C**) in hippocampus of VD (**A**) and AD cases (**B, C**). Arrow and arrowhead show neuropil threads and tangles respectively. Scale bar: 50 um. AD, Alzheimer’s disease; VD, vascular dementia.

**Figure 5:**
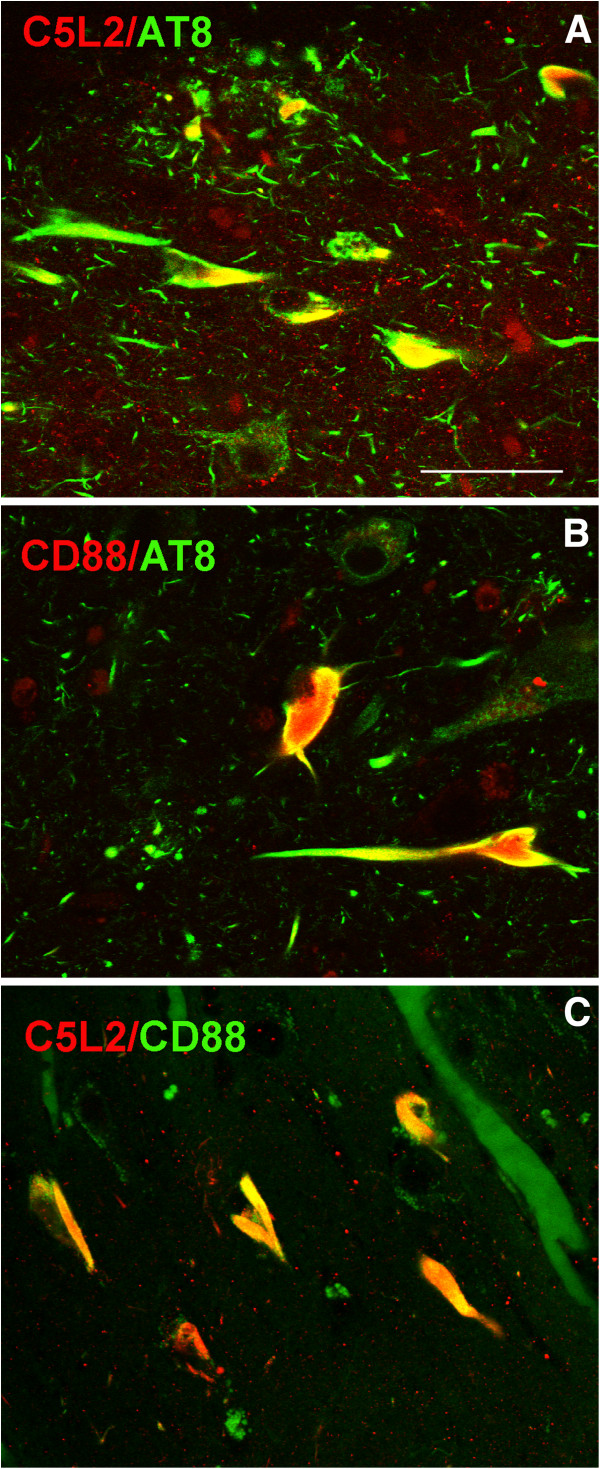
**C5L2 and CD88 colocalize with markers of hyperphosphorylated tau and can be expressed in the same neurofibrillary tangles.** Confocal pictures of hippocampus of AD cases showing colocalization of (**A**) C5L2 (Abcam, anti N1-50, red) and AT8 (green), (**B**) CD88 (Novus anti C300-350, red) and AT8 (green), and (**C**) CD88 (Novus anti C300-350, green) and C5L2 (anti N1-50 red) colocalize in tangles. Scale bar: 50 um. AD, Alzheimer’s disease.

The CD88 distribution in brain was studied with antibodies against the C terminal intracellular (Novus) or N terminal extracellular domains (Abcam) of the human CD88 receptor in the same cases tested with the anti C5L2 antibodies. Both CD88 antibodies reacted similarly, labeling abundantly NFT, NT and DNP mainly in the CA1 and CA2 area of hippocampus of AD cases (Figure 4B, C). In frontal cortex of AD, they labeled similar structures but they were less abundant (data not shown). VD and control cases showed scarce NFT staining in hippocampus (Figure 4A and data not shown). Some light neuronal CD88 staining was also seen in frontal cortex of a few VD and AD cases (data not shown). The degree of immunoreactivity paralleled the severity of the tangle pathology in the AD cases, and was similar to the staining pattern of the C5L2 antibodies (in particular the N1-50 anti C5L2) labeling neurofibrillary pathology. As with the anti C5L2 antibodies, CD88 antibodies also variably stained endothelial cells in blood vessels in control, VD and AD brain (data not shown and Figure 5C). Two additional anti CD88 monoclonal antibodies (clone S5/1 (Serotec) and rabbit monoclonal (BD) showed no reactivity in these same samples (data not shown).

The expression of CD88 also was assessed by Western blot in the same cases as the C5L2 receptor with the Abcam anti C-terminus antibody (Figure 3A). In AD cases the 37 K Mr band was 143% of the control (*P* <0.001). While the Novus anti CD88 did not recognize the denatured CD88 on Western blot, the BD monoclonal anti CD88 also showed increases in the 37 K Mr band in AD cases (data not shown, Table 2).

### C5L2 and CD88 receptors colocalize with hyperphosphorylated tau markers

Immunofluorescent double labeling of C5L2 with early (AT8) (Figure 5A) or mature (PHF-1) (data not shown) tangle markers confirmed co-localization of C5L2 with phosphorylated tau in structures which morphologically appeared to be NFTs, DNPs or NTs in frontal cortex and hippocampus in the AD brain. Similar to C5L2, CD88 immunoreactivity was also found in NFTs and DNP colocalized with AT8 (Figure 5B) and PHF-1 (data not shown). However, not all AT8 or PHF-1 positive neurofibrillary pathology was C5L2 or CD88 positive.

### C5L2 and CD88 receptors colocalize in neurofibrillary tangles

Colocalization of C5L2 and CD88 in human brain tissue was assessed in control, VD and AD cases. These receptors have a low overall sequence homology of 35%. Therefore, in order to avoid any cross reactivity, two antibodies directed against different epitopes with even lower sequence identity were used for double labeling: the N terminus was selected for the C5L2 antibody (N1-50, Abcam) and the C terminus (C300-350, Novus) for CD88. These regions of the two receptors lacked significant overlap (24% and 26% identity respectively for CD88 and C5L2). Confocal images showed that while the receptors could be seen independently expressed, they exhibited a high degree of colocalization in NFTs (Figure 5C). Evaluation of CD88 and C5L2 receptor colocalization resulted in a Mander’s coefficient of 0.66 which is equivalent to 66% colocalization in the hippocampal sections analyzed. The coexpression of the receptors was consistent with the similar patterns of distribution and colocalization with hyperphosphorylated tau markers described above (Figures 1, 2 and 4).

## Discussion

The two identified cell surface receptors for the complement activation peptide, C5a, designated CD88 and C5L2, have been reported to be upregulated in mouse models of several neurodegenerative (inflammatory) diseases, suggesting a role in either disease pathology or modulation or both (reviewed in [22,35]). The results presented here show increased CD88 and C5L2 protein expression in the brain of human AD cases as compared with age-matched controls. CD88 and C5L2 were associated with NFTs (mainly intraneuronal), DNPs, and NTs in the hippocampus and frontal cortex of AD cases. While CD88 and C5L2 were detected in the endothelial cells lining vessel walls, CD88 and C5L2 receptors were predominantly colocalized with both early (AT8) and mature (PHF) tangle markers associated with degenerating neurons.

The detection of a quantitative enhancement of CD88 in AD brain differs from previous studies that showed either similar neuronal expression of CD88 receptor between control and AD samples [12], or a decrease of the CD88 receptor in neuron populations of AD brains compared to controls [31]. These quantitative differences observed in IHC studies could be attributed to differences in tissue processing or in the epitopes recognized by the antibodies. The antibodies in our study were raised against an N terminus epitope similar to, but not identical with, that used by O’Barr and colleagues [12]. In our study, the results were confirmed with an antibody raised against a C terminal CD88 epitope which showed similar staining patterns. In addition, Western blot analysis using brain lysates from the same cases used for IHC also showed an overall increase in reactivity of both the CD88 37 k Mr and 45 k Mr bands in the AD samples compared to controls, thereby validating our IHC data.

The C5L2 receptor distribution at the protein level in the CNS of human samples has not been previously reported, although Northern blot analysis demonstrated mRNA expression in human frontal cortex, hippocampus, hypothalamus and pons [36]. In rat brain, C5L2 was reported to be present constitutively in neurons and astrocytes and also upregulated by noradrenaline correlating with a hypothesized anti-inflammatory role [28]. In a rat model of amyotrophic lateral sclerosis (ALS) C5L2 receptor was upregulated at early stages of the disease in motor neurons [26,37] and was shown to be colocalized with ubiquitinated intracellular aggregates which are characteristic of ALS [37]. In the present work, C5L2 was associated with NFTs, prevalent in AD brain. Protein levels were increased in hippocampal lysates from AD brains compared to controls. These results were validated by utilizing two distinct cohorts of AD and control samples as well as antibodies to two different C5L2 epitopes. Preabsorption of the anti N1-23 antibody with the N1-23 peptide (N-terminus of C5L2) showed a loss of immunostaining in cortex and hippocampus and a blockade of reactivity of the 37 k Mr band in Western blots confirming that the reactivity detected was indeed against C5L2.

The role of C5a receptors in neurons, microglia and astrocytes in neuroinflammatory diseases (for review see [22]) is not clear. Some reports have suggested that C5a induces neuroprotective pathways in some scenarios [12,38], and enhanced recruitment of phagocytes to plaques could be postulated to facilitate clearance of plaques and cell debris [25,39]. While both beneficial and detrimental roles are not mutually exclusive, support for an overriding detrimental role for CD88 has been provided by the improvement in pathology and clinical symptoms obtained in animals models of AD, Huntington’s disease (HD) and ALS treated with specific antagonists for this receptor (PMX53 and PMX205) [25-27], as well as in ischemic stroke [11]. In murine AD models, PMX205 was effective in reducing plaques, reactive glia, and improving neuronal integrity as well as cognition [25]. While the exact mechanism is not known, the protective effects of the antagonist could be due to the blockade of C5a binding to CD88 in glia, although an interaction of PMX205 with a neuronal CD88 is also possible. In the Tg2576 and Arc48 mouse models of AD, CD88 receptors were shown to be present in microglia polarized towards plaques and were upregulated in parallel with plaque and glia pathology [24]. The lack of glial CD88 detection in our study with human tissue could be due to the differential ability of the antibodies used to detect glial CD88 in fixed tissue and/or a lower degree of inflammation in the human AD brain at the time of death relative to that in the inflamed brains reported by others [40,41]

The presence of C5aR in neurons suggest their involvement in functional roles that may differ from those in glia and may also be different according to neuronal cell types and/or stages of development. Several putative roles have been attributed to the neuronal C5aR, such as cytoskeletal plasticity, induction of adhesion molecules or neurotrophins and clearance of anaphylotoxins (for reviews see [22,35,42]). CD88 has also been proposed to have a role in the development of cerebellum [43] and to act as a guidance cue for granule cells [44]. In contrast, C5aR might be involved in apoptosis in neurons (15). Recently, C5a was shown to be generated by CNS neurons and to induce neuronal apoptosis upon ligation of CD88 in murine systems [11]. However, the role of CD88 and C5L2 prior to their colocalization accumulated in NFTs (as seen in our study) is unknown. CD88 and C5L2 have been previously shown to colocalize in human neutrophils, with CD88 mainly expressed extracellularly and C5L2 mainly intracellularly. After ligand binding, the CD88 receptors were internalized, and both (CD88 and C5L2) colocalized and associated with ß arrestin. It was postulated that the C5L2 receptor can negatively modulate neutrophil CD88 receptor signaling [21] reflecting an anti-inflammatory function. C5L2 has also been postulated to have other roles, such as decoy receptors and could even exert a proinflammatory function (for a review [45]). However, at the stage where both C5L2 and CD88 colocalize with NFTs, these receptors are probably in a non-functional state.

The accumulation of the C5a receptors observed in AD could be due to increased synthesis and/or decreased degradation. TNFα, a proinflammatory cytokine released by microglia and astrocytes [46], is increased in AD brains [47] and has been shown to mediate the upregulation of CD88 receptor mRNA and protein in neurons *in vivo*[48]. Region specific increases in CD88 mRNA that reached statistical significance during aging and with AD were shown by microarray analysis of a relatively large cohort of young, older and AD individuals [49]. However, the observed increased levels and association of both CD88 and C5L2 with tangles is also consistent with a common altered pathway of degradation. Seven transmembrane receptors, like CD88, recruit ß-arrestin, and, upon internalization, the receptor and ß-arrestin are ubiquitinated leading to degradation (reviewed in [50]). In PHF’s, tau is also ubiquitinated [51,52]. In AD a decreased activity of the ubiquitin proteasome system (UPS) has been observed, and PHF-tau was shown to inhibit UPS mediated protein degradation [53]. Thus, it is possible that the proteasome dysfunction observed in AD can cause tau, C5L2 and CD88 accumulation. In addition, deficits in autophagy, another mechanism of protein clearance important for removal of aggregates and misfolded proteins, are associated with AD [54,55]. Such defects could also contribute to decreased turnover of CD88 and C5L2.

In summary, levels of the receptors for the complement activation fragment C5a, CD88 and C5L2 are elevated in AD brain. Their colocalization with NFTs suggests that this accumulation could predominantly be a consequence of altered turnover of these receptors, rather than an increase in synthesis to contribute to, or compensate for, an inflammatory environment. While these findings are also consistent with a role of these receptors in neuron degeneration, they do not rule out a role for glial CD88 in AD pathology. Given the potential therapeutic value of inhibiting CD88 function to prevent or slow progression of AD as demonstrated in murine models of AD, further study of these receptors in the brain is warranted.

## Competing interests

The authors declare that they have no competing interests.

## Authors’ contributions

MF contributed to the design of the study, performed immunohistochemistry, immunoblotting and quantification and analysis and prepared the draft of the manuscript. SM and SC contributed to the conceptual design, immunoblotting experiments and their densitometric analysis, and preparation of the manuscript. AT contributed to the design of the study and to the preparation of the manuscript. All authors have read and approved the final version of the manuscript.

## Supplementary Material

Additional file 1: Figure S1 C5L2 immunostaining of AD brain using C5L2 N-1-23 antibody (brown) and counterstained with cresyl violet showing neurofibrillary tangle labeling in hippocampus. Scale bar 50 um. Click here for file
