# Supplementary figures and images for: Complement activation fragment C5a receptors, CD88 and C5L2, are associated with neurofibrillary pathology
Source: J Neuroinflammation. 2013 Feb 8;10:25. doi: 10.1186/1742-2094-10-25 (PMC3605123; doi:10.1186/1742-2094-10-25)

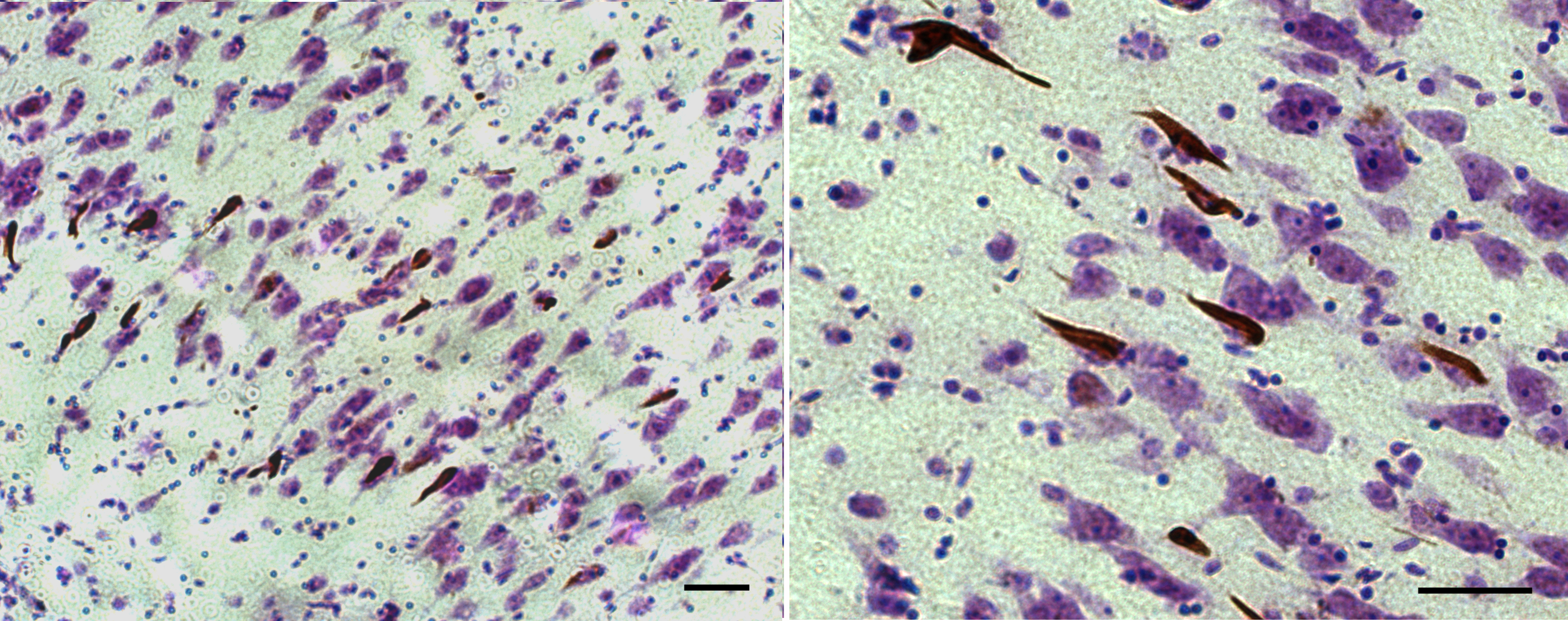

Supplement: Additional file 1: Figure S1 — C5L2 immunostaining of AD brain using C5L2 N-1-23 antibody (brown) and counterstained with cresyl violet showing neurofibrillary tangle labeling in hippocampus. Scale bar 50 um. [file 1742-2094-10-25-S1.tiff]
